# Multifunctional Flax Fibres Based on the Combined Effect of Silver and Zinc Oxide (Ag/ZnO) Nanostructures

**DOI:** 10.3390/nano8121069

**Published:** 2018-12-19

**Authors:** Sofia M. Costa, Diana P. Ferreira, Armando Ferreira, Filipe Vaz, Raul Fangueiro

**Affiliations:** 1 Centre for Textile Science and Technology (2C2T), University of Minho, 4800 Guimarães, Portugal; sofiamcosta@det.uminho.pt (S.M.C.); rfangueiro@dem.uminho.pt (R.F.); 2 Center of Physics, University of Minho, 4710-057 Braga, Portugal; armando.f@fisica.uminho.pt (A.F.); fvaz@fisica.uminho.pt (F.V.); 3 Department of Mechanical Engineering, University of Minho, 4800 Guimarães, Portugal

**Keywords:** smart and multifunctional materials, cellulosic fibres, silver and zinc oxide nanoparticles, piezoresistive response, antibacterial effect, hydrophobicity

## Abstract

Cellulosic fibre-based smart materials exhibiting multiple capabilities are getting tremendous attention due to their wide application areas. In this work, multifunctional flax fabrics with piezoresistive response were developed through the combined functionalization with silver (Ag) and zinc oxide (ZnO) nanoparticles (NPs). Biodegradable polyethylene glycol (PEG) was used to produce AgNPs, whereas ZnONPs were synthetized via a simple and low-cost method. Flax fabrics with and without NPs were characterized by Ground State Diffuse Reflectance (GSDR), Field Emission Scanning Electron Microscopy (FESEM), Energy Dispersive Spectroscopy (EDS)**,** X-ray Diffraction (XRD), Attenuated Total Reflectance-Fourier Transform Infrared Spectroscopy (ATR-FTIR), and Thermogravimetric analysis (TGA). After creating a conductive surface by flax functionalization with AgNPs, ZnONPs were synthetized onto these fabrics. The developed fibrous systems exhibited piezoresistive response and the sensor sensitivity increased with the use of higher ZnO precursor concentrations (0.4 M). Functionalized fabrics exhibited excellent antibacterial activity against Gram-negative (*Escherichia coli*) and Gram-positive (*Staphylococcus aureus*) bacteria, higher hydrophobicity (WCA changed from 0^0^ to >100^0^), UV radiation resistance, and wash durability. Overall, this work provides new insights regarding the bifunctionalization of flax fabrics with Ag/ZnO nanostructures and brings new findings about the combined effect of both NPs for the development of piezoresistive textile sensors with multifunctional properties.

## 1. Introduction

Over the last few years, the research and development of fibre-based smart materials that are capable of detecting and responding to external stimuli has increased dramatically. These materials can be applied in several areas including healthcare, electronics, sports, water treatment, military and aerospace [1,2]. The modification of cellulosic fibres’ surfaces with nanostructures allows the possibility to build sensors (e.g., piezoresistive sensors) directly onto the fabrics, creating wearable electronic devices [3,4]. Besides sensing properties, these fibre-based materials can also present multiple functions such as ultraviolet (UV) protection, flame retardancy, hydrophobicity/self-cleaning, and antibacterial effect, depending on the surface functionalization. Nanoparticles (NPs) are very attractive materials to be used in the development of functional fibrous systems, due to their higher surface area and exclusive optical, electrical, and catalytic properties [5,6,7]. Silver (Ag) and zinc oxide (ZnO) NPs exhibit numerous interesting properties, such as antibacterial effect, UV-blocking, piezoresistive properties, electrical conductivity, and photocatalytic activity, being widely used to functionalize different cellulosic fibres [6,8,9,10,11]. Hence, the combined effect of these NPs can be extremely useful for multifunctional materials development.

Despite the strong research in this area [12], there are few problems related with sustainability and mass production that are still not well addressed. Thus, there is a huge need to explore and improve the processes sustainability, the cost and easiness of experimental methodologies, the industrial scalability, and the systems durability/washability. Taking into account the materials sustainability, natural cellulosic fibres (NCF) have emerged as valuable alternatives to replace synthetic ones, leading to a reduction in environmental pollution [13,14]. Moreover, NCF present numerous advantages, including a high abundance, low-cost, biodegradability, biocompatibility, low-weight, and good mechanical properties, making them very suitable materials to develop environmentally friendly fibrous systems. Among NCF, flax fibres are considered as one of the most promising and valuable, due to their high specific mechanical properties, being one of the most used as reinforcement in composite materials [13,14,15,16]. Besides the use of green materials, avoiding extremely high temperatures and choosing non-toxic and biodegradable solvents is also of particular importance.

In the present study, the potential of Ag/ZnO nanostructures for multifunctional fibrous systems development was evaluated, taking into consideration the sustainability of the materials and methodologies involved. The development of piezoresistive sensors requires a conductive surface, which can be achieved by the functionalization of flax fabrics with AgNPs, since these NPs have electrical properties. In this way, AgNPs were synthetized using an eco-friendly and very easy methodology with polyethylene glycol (PEG) (as reducing and stabilizing agent). Afterwards, the *in-situ* synthesis of ZnONPs was performed onto AgNPs-treated flax fabric, using zinc acetate as precursor, with water as solvent, and a minimal concentration of sodium hydroxide (NaOH). At all stages, the processes scalability has always been considered, as well as the methodologies costs. All of the developed samples were characterized by Ground State Diffuse Reflectance (GSDR), Field Emission Scanning Electron Microscopy (FESEM), Energy Dispersive Spectroscopy (EDS), X-ray Diffraction (XRD), Attenuated Total Reflectance-Fourier Transform Infrared Spectroscopy (ATR-FTIR), and Thermogravimetric analysis (TGA). Finally, the fabrics multiple properties were evaluated, including the electrical conductivity, piezoresistive behaviour, antibacterial effect, hydrophobicity, UV resistance, and washability.

## 2. Materials and Methods

### 2.1. Materials

The flax fabrics were supplied by RCS (Braga, Portugal) with an areal mass of 315 g/m^2^. Silver nitrate (AgNO_3_) and PEG Mr ~200 were purchased from Sigma Aldrich. Zinc acetate dehydrated (Zn(CH_3_COO)_2_·2H_2_O) and NaOH were obtained from Akzo Nobel. An UV blacklight lamp was used (15 W) from HQ^TM^.

### 2.2. Flax Fabrics Pretreatment

Flax fabrics (4 cm × 4 cm) were washed with 5 % (*v*/*v*) of non-ionic detergent at 80 °C for 30 min to remove impurities (waxes, fats, etc). The fabrics were further cleaned in distilled water at 70 °C for 30 min and then dried at 100 °C for 10 min.

### 2.3. Functionalization of Flax Fabrics with NPs

#### 2.3.1. Green Synthesis and Deposition of AgNPs onto Flax Fabrics

AgNPs were synthetized and incorporated onto flax fabrics accordingly with the method that was previously described by Ferreira et al. [5]. Briefly, a 0.1 M AgNO_3_ and PEG200 solution was prepared and kept under stirring for one hour at 25 °C. The solution colour change to yellow indicates the reduction of Ag ions into Ag metal (Ag^0^). After this, the fabrics were impregnated with this solution and kept overnight. The dip-pad-dry method was used to improve the incorporation of the AgNPs into the fabrics. The samples were dried at 100 °C for 20 min, washed with water, and dried again at 100 °C for 6 min.

#### 2.3.2. In-Situ Synthesis of ZnONPs onto AgNPs-Treated Flax Fabrics

ZnONPs were synthetized following the method that was described in [17], with few modifications. Briefly, AgNPs-treated flax samples were immersed on aqueous solutions of Zn(CH_3_COO)_2_·2H_2_O with different concentrations (0.01 M, 0.05 M, 0.1 M, 0.2 M and 0.4 M). The solutions were heated to 50 °C under constant stirring (1 h), and 10 mL of NaOH (1 M) was added (1 mL/min). Finally, Ag/ZnO-treated flax samples were removed from the solution and heated to 100 °C for 6 h. The samples will be described accordingly with the precursor (Zn(CH_3_COO)_2_·2H_2_O) solution concentration under use (example: flax + AgNPs + xMZnONPs, x = 0.01, 0.05, 0.1, 0.2 or 0.4 M).

### 2.4. Flax Fabrics Samples Characterization

#### 2.4.1. Ground State Diffuse Reflectance (GSDR)

The fabric samples’ GSDR spectra were recorded in the 250 to 700 nm wavelength range, using a Spectrophotometer UV 2501PC Shimadzu. Each sample was analysed in three different places to ensure homogeneity. The remission function (F(R)) was calculated accordingly with the Kubelka-Munk equation:

(1) $$F\left(R\right)={\frac{\left(1-R\right)}{2R}}^{2}=\frac{K}{S},$$

where *K* is the absorption coefficient and *S* is the dispersion coefficient.

#### 2.4.2. Field Emission Scanning Electron Microscopy (FESEM) and Energy Dispersive Spectroscopy (EDS)

The samples surface morphology was analysed by FESEM using a NOVA 200 Nano SEM from FEI Company (Hillsboro, OR, USA). All of the samples were coated with a very thin film (20 nm) of Gold (Au)-Palladium (Pd) before the experiment. Images were taken in topographic mode with an accelerated voltage of 10 kV. The EDS technique (Hillsboro, OR, USA) coupled to FESEM was performed to evaluate the elemental composition of the samples, using an EDAX Si (Li) detector with 15 kV of acceleration voltage. The solution of PEG containing AgNPs was analysed in transmission electron mode and the samples were installed in Cu-C grids by immersion in the solution. After this, the samples were analysed with an acceleration voltage of 15 kV, using a scanning transmission electron detector (STEM) (Hillsboro, OR, USA). A Zeiss Ultra 55 FESEM (Hillsboro, OR, USA) was used to observe the morphology of the samples using an acceleration voltage of 3 kV. Three samples for each condition was evaluated. 

#### 2.4.3. X-ray Diffraction (XRD)

The crystallinity of the NPs was evaluated using a Bruker D8 Discover diffractometer, operated at a voltage of 40 kV and a current of 40 mA with Cu-K_β_ radiation. Data were collected for 2θ values ranging from 10° to 90°.

#### 2.4.4. Attenuated Total Reflectance-Fourier Transform Infrared Spectroscopy (ATR-FTIR)

Chemical composition of the fabric samples was studied by ATR-FTIR analysis using an IRAffinity-1S, SHIMADZU equipment (Kyoto, Japan). Each spectrum was obtained in transmittance mode using a diamond ATR crystal cell by accumulation of 45 scans with a resolution of 8 cm^−1^ from 400 to 4000 cm^−1^. 

#### 2.4.5. Thermogravimetric Analysis (TGA)

TGA analysis was performed in order to estimate the amount of NPs incorporated onto flax fabrics using a STA 700 SCANSCI. Untreated and treated fabrics were subjected to a heating process from room temperature to 600 °C under nitrogen flow and with a heating rate of 20 °C/min. The percentage of NPs that were deposited on the flax fabric was calculated using the following system of equations:

(2) $$\{\begin{array}{c} NPs\;\%+flax\;fabric\;\%\left(\frac{flax\;fabric\;residues\%}{100}\right)=sample\;total\;residues\;\%;\\ NPs\;\%+flax\;fabric\;\%=100\%\; \end{array}$$

Considering that the nanoparticles have residues of 100% and the sample with NPs after thermal treatment is composed of NPs and flax fabric residue. The samples were tested in triplicate in order to diminish the associate error to natural fibres (heterogeneous surface).

### 2.5. Multifunctional Properties Evaluation

#### 2.5.1. Resistivity Measurements and Electromechanical Characterization

**Figure 1:**
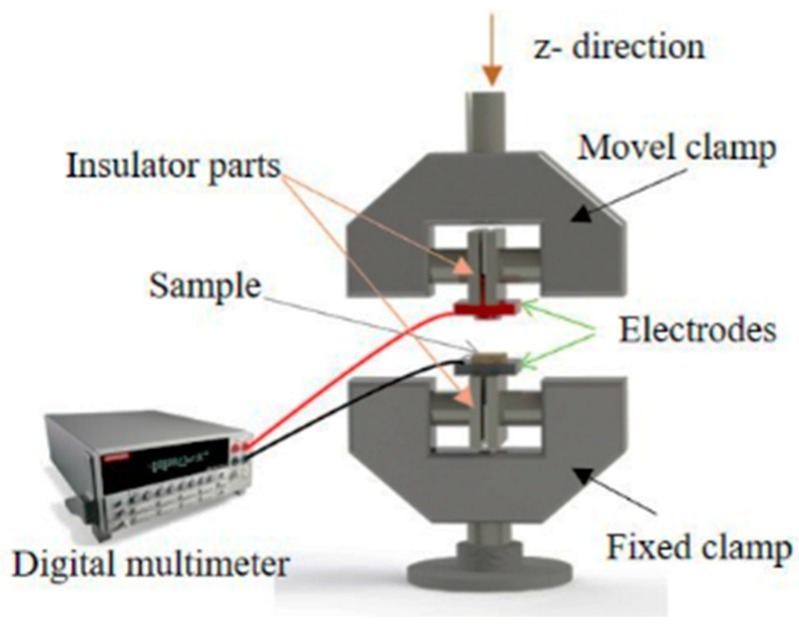
Schematic representation of the experimental configuration of the clamps for the compression experiments with simultaneous electrical measurements for electro-mechanical response evaluation of the samples.

The electrical conductivity of the flax samples depends on the formation of a continuous conductive network in the insulating matrix. In order to evaluate pressure sensing properties of flax fabric, the samples were subjected to a compression of 20%. The sensing experiments were performed in a compression configuration using a Shimadzu-AG-IS universal testing machine with a load cell of 500 N, a z-deformation of 0.5 mm, and a compression velocity of 4 mm·min^−1^ on samples with a diameter of 10 mm, during 10 cycles. The sensitivity average value was calculated from four different measurements for each sample. The electro-mechanical tests were performed by measuring the electrical resistance of the sample, through the electrodes placed into the clamps (Figure 1). The measurement of the surface resistance change ∆R/R_0_ in each mechanical experiment was obtained from the measurement of the electrical resistance, with a Keithley 2700 digital multimeter. 

Before that, the electrical resistivity of the flax samples was measured by the two-wire method, where the voltage was applied with a range from −10 V to 10 V, step of 1, and the current measured by a Keithley 487 picoammeter/voltage source. All of the measurements were performed in direct current (DC) mode, at room temperature. The electrical resistivity (*ρ*) was calculated by:

(3) $$\rho =R\frac{A}{L}$$

where *R* is the electrical resistance, A is the area of the electrode (6 × 1 mm^2^), and L is the distance between the electrodes (2 mm). The electrical conductivity is given by the inverse of the electrical resistivity (σ = 1/*ρ*).

The sensing properties of the flax samples was quantified by the gauge factor (*GF*), which is defined as the ratio between the variation in the electrical resistance and the mechanical deformation applied [18]:

(4) $$\frac{\Delta R}{R_{0}}=GF\frac{\Delta l}{l_{0}}$$

where *R*_0_ is the steady-state material electrical resistance before deformation, ∆*R* is the resistance change that is caused by the mechanical deformation, *l*_0_ is the initial thickness of the flax sample, and Δl the thickness variation. Three replicates of each condition were evaluated. 

#### 2.5.2. Antibacterial Tests

The antibacterial activity of the solutions containing NPs and the functionalized flax fabrics was assessed, following the Agar Well Diffusion Method [19] and the JIS L 1902–Halo Method [20], respectively, against Gram-negative (*Escherichia coli*) and Gram-positive (*Staphylococcus aureus*) bacteria. Firstly, two different inoculums containing each bacteria tested were prepared using nutrient broth (NB) and incubated for 12–18 h at 37 °C. Regarding the agar well diffusion method, nutrient agar (NA) was prepared, autoclaved, and applied onto petri dishes until agar solidification. Subsequently, 100 µL of inoculum with a concentration of 1 × 10^6^ cells/mL was spread over the entire agar surface. The agar medium was punched to create five wells and each well was filled with different solutions, in equal amounts. The agar dishes were incubated for 24 h at 37 °C [19]. According to the JIS L 1902–Halo method, 0.33 mL of inoculum containing 1 × 10^7^ cells/mL was added to 4.67 mL of NA, and finally, distributed uniformly onto sterilized petri dishes. After agar solidification, square flax fabric samples of 1 cm × 1 cm were placed over the agar dishes and incubated for 24 h at 37 °C [20]. After samples incubation, the antibacterial activity was evaluated by the halo formation (inhibition zone) around the edges of the samples, for both methods. The appearance of the inhibition zone indicates the absence of bacteria growth, demonstrating the samples’ antibacterial effect. The diameter of inhibition zones formed around the edges of the samples was measured in order to infer about the potency of antibacterial activity. Three replicates of each condition were tested.

#### 2.5.3. Water Contact Angle Measurement

The water contact angle (WCA) was determined using a Contact Angle System (dataphysics) coupled to a high-resolution camera. A volume of 5 µL of distilled water was dispensed from the syringe onto the sample’s surface. Each sample was measured in 10 different places and the results were averaged to obtain both mean and standard deviations.

#### 2.5.4. UV Radiation Resistance and Washability

In order to evaluate the UV resistance ability of functionalized flax samples, they were placed under UV light (distance = 10 cm) for a total period of 80 h and colour measurements were assessed in distinct time points with a Datacolor spectrophotometer using the Cielab coordinates D65/10 software (Lucerne, Switzerland). The necessary CieLab parameters were determined: L* is the lightness from black (0) to white (100); a* indicates if the sample is redder (positive values) or greener (negative values); and, b* indicates if the sample is yellower (positive values) or bluer (negative values). Finally, the overall/total difference (∆E*) between the evaluated samples (exposure to different times of UV radiation) and the initial ones (no exposure to UV radiation) were calculated using the following equation:

(5) $$\Delta E={\left[{\left(\Delta L*\right)}^{2}+\;{\left(\Delta b*\right)}^{2}+{\left(\Delta a*\right)}^{2}\right]}^{1/2}$$

To evaluate the wash durability of the Ag/ZnO NPs active coating onto flax fabric, the samples were placed in contact with distilled water and centrifuged at 200 rpm for 100 h, continuously. This procedure mimics the domestic washing programmes accordingly with the rpm values that were described in the standard ISO6330–Textiles, Domestic washing and drying, procedures for textile testing. In this standard, the agitation speed could range from 119 rpm to 179 rpm (delicate and durable press parameters, respectively). The main goal of this test was to show that the NPs are fixed onto the fabrics even with a continuous contact with water under rotation and not to detach the NPs from the fabrics. Therefore, a small volume of the washing solution was collected at specific time points (15 min, 30 min, 1 h, 2 h, 24 h, 72 h, and 100 h) and the absorbance was measured (spectrophotometer UV-1800, Shimadzu, wavelength range: 200–800 nm).

### 2.6. Statistical Analysis

Statistical analysis was performed using a GraphPad prism (version 6.0). The Shapiro–Wilk test was used to evaluate the normal distribution of the samples. A parametric ANOVA was performed to evaluate the differences between the samples. Data are presented by mean ± SD. Statistical significance was considered if *p* < 0.05.

## 3. Results and Discussion

### 3.1. Synthesis and Characterization of Flax Fabrics Functionalized with AgNPs and ZnONPs

As mentioned before, the main goal of this work was to functionalize cellulosic fibres, namely flax fabrics with Ag/ZnO hierarchical nanostructures to introduce multiple new properties onto these fabrics. In addition, always considering the sustainability of the processes, the methodologies scalability, and the durability. Initially, Ag NPs were used to develop a conductive surface and the ZnO NPs to introduce a better piezoresistive response. Overall, both NPs presented other functionalities that will be described further, such as: antibacterial activity and higher hydrophobicity. In this way, AgNPs were synthesized by an eco-friendly method that was previously described by authors [5]. In this method, the non-toxic and biodegradable PEG polymer was used as a reducing and stabilizing agent. The PEG hydroxyl groups are responsible for the progressive reduction of Ag^+^ ions into Ag^0^, as demonstrated by the following equation [5]:

(6) $${\mathrm{CH}}_{2}{\mathrm{CH}}_{2}\mathrm{OH}+{\mathrm{Ag}}^{+}\to {\mathrm{CH}}_{2}\mathrm{CHO}+{\mathrm{Ag}}^{0}$$

PEG also acts as stabilizer and dispersing agent, because this polymer covers the surface of AgNPs, preventing their aggregation. Furthermore, hydrogen bonds can be formed between PEG groups and cellulose hydroxyl groups from flax, improving the incorporation of AgNPs onto the flax fabric. After this, in-situ synthesis of ZnONPs was performed onto AgNPs-treated flax fabrics, using a simple and low-cost method. In this process, NaOH acted as the reducing agent of zinc acetate (precursor) to produce ZnONPs, accordingly with the following reactions [17]:

(7) $$\begin{array}{c} \mathrm{Zn}{\left({\mathrm{CH}}_{3}\mathrm{COO}\right)}_{2}\cdot 2H_{2}O+2\mathrm{NaOH}\to \mathrm{Zn}{\left(\mathrm{OH}\right)}_{2}+2{\mathrm{CH}}_{3}\mathrm{COONa}+2H_{2}O\\ \mathrm{Zn}{\left(\mathrm{OH}\right)}_{2}+2H_{2}O\to \mathrm{Zn}{\left(\mathrm{OH}\right)}_{4}^{2-}+2H^{+}\\ \mathrm{Zn}{\left(\mathrm{OH}\right)}_{4}^{2-}\to \mathrm{ZnO}+H_{2}O+2{\mathrm{OH}}^{-} \end{array}$$

Several precursor (zinc acetate) concentrations ranging from 0.01 M to 0.4 M were used in order to infer about their influence in the NPs synthesis, deposition, and dispersion, as well as in the final properties, including piezoresistive response, antibacterial effect, hydrophobicity, and UV resistance.

#### 3.1.1. Ground State Diffuse Reflectance (GSDR)

**Figure 2:**
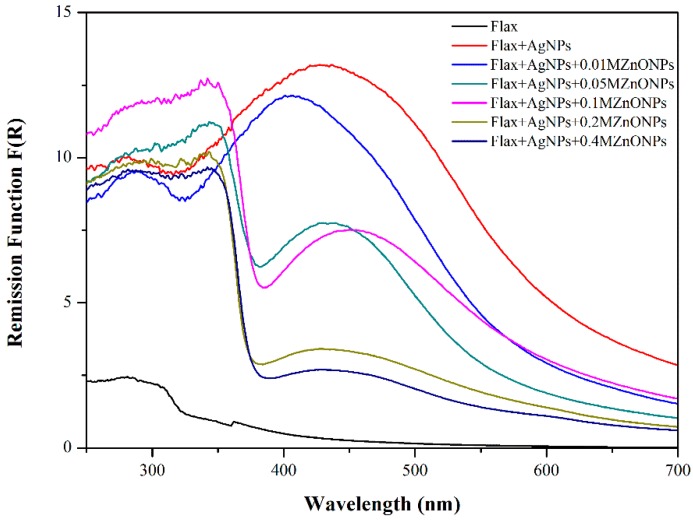
Ground State Diffuse Reflectance (GSDR) spectra of flax fabric treated with silver nanoparticles (AgNPs) and different concentrations of zinc oxide nanoparticles (ZnONPs).

GSDR was used to evaluate the fabrics functionalization with both NPs. The Kubelka–Munk remission function of flax fabrics functionalized with AgNPs and different concentrations of ZnONPs are shown in Figure 2. Each spectrum represents the mean of R values that were obtained for the measurement in three different places of samples.

Flax fabrics functionalized with AgNPs exhibit a peak at approximately 437 nm, which indicates the surface plasmon resonance (SPR) band formation of AgNPs. In the flax fabrics’ spectra treated with both Ag and ZnO NPs it is visible the appearance of a new absorption band at UV region (~380 nm), characteristic of ZnONPs. Furthermore, the sharp shape of the ZnONPs absorption band indicates that these NPs are monodispersed [21,22]. Nevertheless, as the concentration of ZnONPs precursor increases (from 0.05 M to 0.4 M), there is a decrease in the intensity, broadening, and shifting of the AgNPs absorption band. This phenomenon can be related with the increasing size of NPs (as will be shown with FESEM analysis), with the highest quantity of ZnONPs as compared with AgNPs and with the presence of AgNPs agglomerates/nanoclusters [5,23]. At the same time, with the second process (ZnONPs functionalization), AgNPs that are not so strongly attached to the fabric surface are lost, decreasing the absorption band intensity. The in-situ synthesis of ZnONPs onto flax fabric is performed after synthesis and deposition of AgNPs onto flax. Firstly, the AgNPs are incorporated onto the flax fabric, the samples are washed and dried. After this, the flax fabrics with the AgNPs are subjected to the in-situ synthesis of ZnONPs in solution and using NaOH as reducing agent. It is normal that during this process, the AgNPs that are not so strongly attached to the surface are lost due to the use of NaOH and also due to the stirring process, which is why the intensity of the plasmon band decreases. However, the plasmon band is still there and the fabrics still present the strong yellow colour correspondent to the AgNPs presence. Moreover, with the minimum concentration of ZnONPs precursor (flax + AgNPs + 0.01 MZnONPs), only the absorption band of AgNPs is visible, indicating that this concentration is too low to produce ZnONPs.

#### 3.1.2. Field Emission Scanning Electron Microscopy (FESEM) and Energy Dispersive Spectroscopy (EDS)

**Figure 3:**
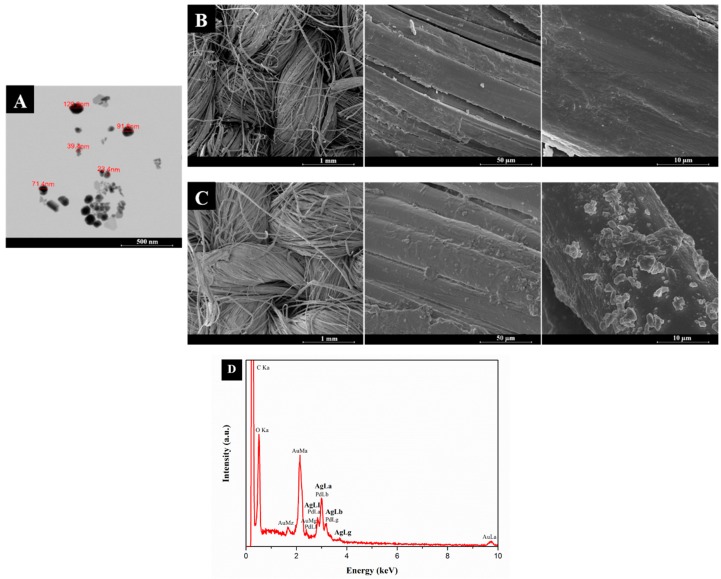
Scanning transmission electron detector (STEM) micrograph of the AgNPs synthesized in polyethylene glycol (PEG) solution with magnifications of 500 nm (**A**). Field Emission Scanning Electron Microscopy (FESEM) images of untreated flax (**B**) and flax + AgNPs (**C**) with magnifications of 1 mm, 50 µm and 10 µm (from the left to the right). The images are in topographic mode. Energy Dispersive Spectroscopy (EDS) spectrum of flax fabric treated with AgNPs (**D**).

The surface morphology of untreated and AgNPs-treated flax fabrics was further evaluated by FESEM and EDS analyses (Figure 3).

**Figure 4:**
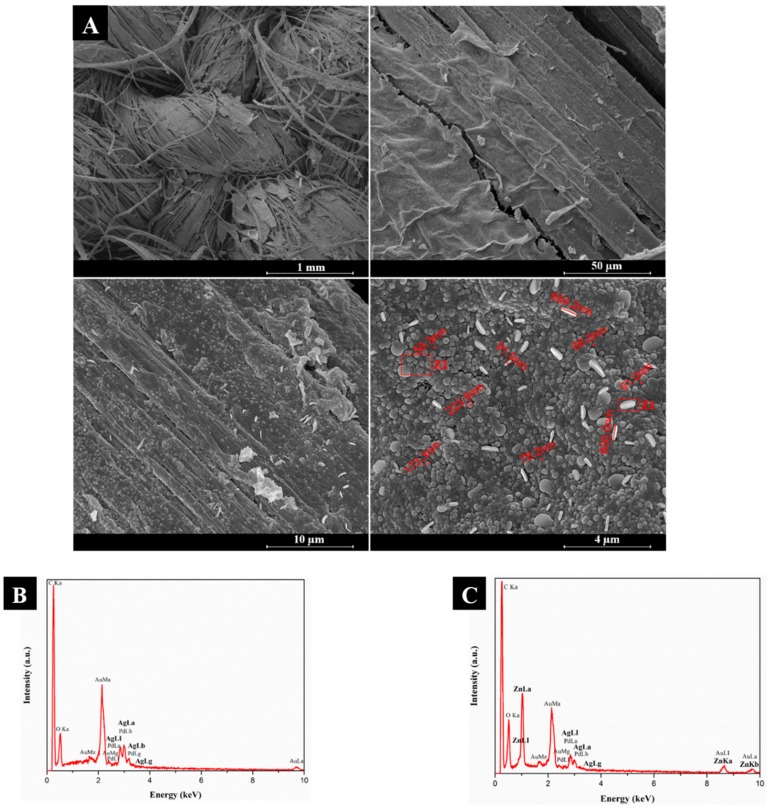
FESEM images of flax fabric functionalized with both AgNPs and ZnONPs (**A**). Magnifications of 1 mm, 50 µm, 10 µm, and 4 µm (from the left to the right). EDS spectra of Z1 zone (**B**) and Z2 zone (**C**).

Figure 3A presents the STEM analysis of the AgNPs that were synthesized using the PEG solution. This image reveals the successful synthesis of the NPs, with sizes ranging from 23.4 to 120.9 nm. After the in-solution synthesis, the NPs were incorporated onto the flax fabric, as can be seen in Figure 3C. The presence of the AgNPs onto the flax surface is visible when compared with the untreated flax fabric showed in Figure 3B. Besides the presence of AgNPs onto the flax fabric, and the presence of an excessive quantity of PEG coating the flax surface is also notable. The use of PEG is very useful in the synthesis process due to their dispersive character. However, during the ZnONPs in-situ synthesis, the excess of PEG is eliminated, as can be seen in Figure 4A. EDS spectra (Figure 3D) shows the presence of several peaks, namely those of elemental carbon and oxygen, which are the main constituents of NCF. An additional strong peak appeared at 2.99 keV, which can be attributed to silver [5,9,24], confirming the presence of Ag onto the flax fabric surface. 

After in-situ synthesis of ZnONPs onto AgNPs-treated flax fabric, FESEM and EDS analyses (Figure 4) were performed to evaluate the surface morphology and elemental composition of the samples, respectively. 

Figure 4 clearly demonstrates the presence of NPs on the surface of flax fabrics, showing a dense and uniform deposition of Ag and ZnO NPs. Moreover, two distinct morphologies of NPs can be seen: spheres and platelets, which can be attributed to ZnONPs and AgNPs, respectively, as confirmed by EDS analysis that was performed specifically in each zone: Z1 (platelets: Figure 4B) and Z2 (spheres: Figure 4C). As shown in Figure 4A, spherical particles have sizes ranging from 58.3 to 223.9 nm, while platelets present diameter in the range from 600 to 684.2 nm and thickness of 91 nm. These results suggest that ZnONPs synthesis process, namely the use of temperature, influences the AgNPs shape, which changed from relatively spherical (Figure 3) to platelets (Figure 4). It has been described that temperature could have a key role in the formation, growth, size, and shape of AgNPs. In fact, Jiang et al. [25] demonstrated that the increase of reaction temperature promoted an increase in AgNPs size. Moreover, the authors reported that increasing temperature led to a reduction in quantity of particles with spherical shape and an increase of platelets particles, which is in agreement with the results that are shown in this work (Figure 4A). The increase in NPs size is probably due to the fusion growth process of small particles into larger ones, which is promoted by the heating process [25]. In this way, the temperature that is used in ZnONPs synthesis (50 °C) could be responsible for the formation of AgNPs with increased size and platelets shape.

In EDS analysis (Figure 4C), besides the presence of Ag related peaks, there is also evidence of strong signals of Zn and O, thus confirming the presence of ZnONPs onto AgNPs-treated flax fabrics. Three new peaks appeared at 1.03, 8.67, and 9.69 keV that are attributed to Zn. These results are concordance with previous studies and confirm the formation of ZnONPs [26].

#### 3.1.3. X-ray Diffraction (XRD)

**Figure 5:**
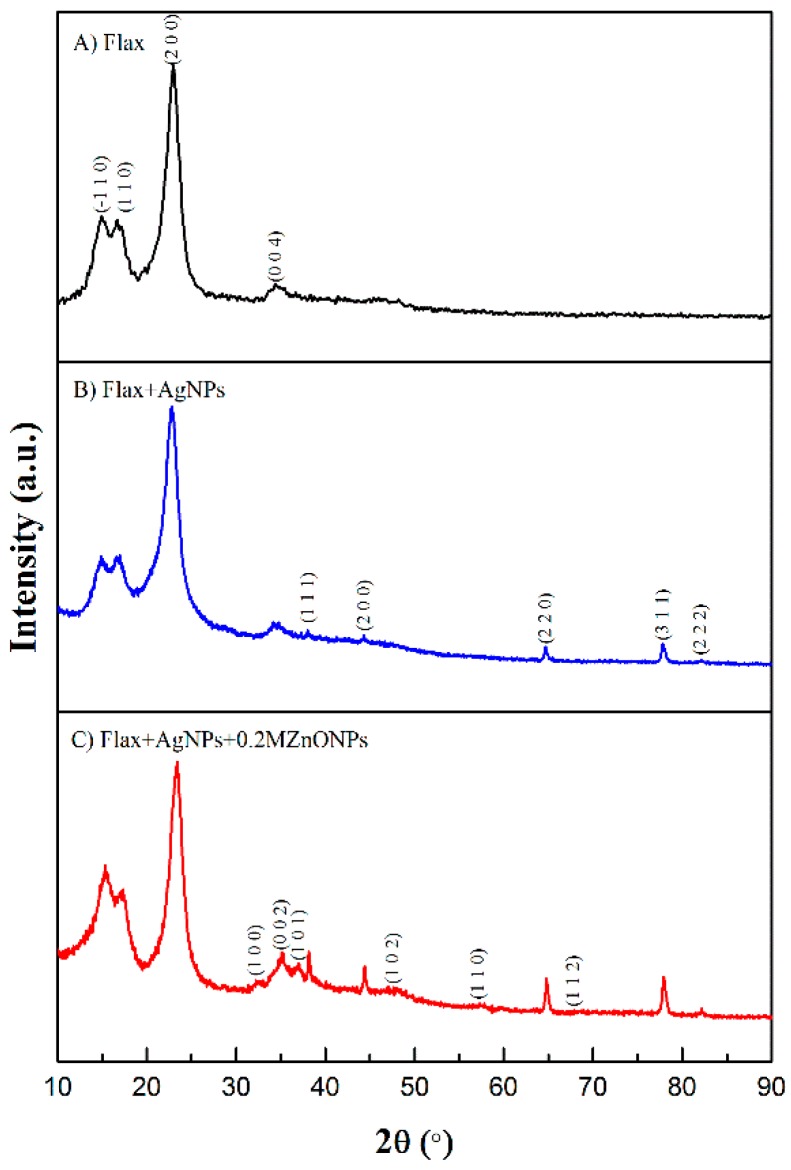
XRD spectra of flax fabric: untreated (**A**), treated with AgNPs (**B**) and treated with Ag and ZnO NPs (**C**).

Figure 5 shows the XRD pattern of untreated flax fabric (A), AgNPs-treated flax fabric (B), and Ag/ZnONPs-treated flax fabric (C).

Flax fabric presents four diffraction patterns that are located at 2θ = 14.92°, 16.68°, 22.96°, and 34.41°, which correspond to the (−1 1 0), (1 1 0), (2 0 0), and (0 0 4) planes, respectively, of crystalline domains of cellulose I [17], the main constituent of NCF. Flax fabric treated with AgNPs clearly demonstrates the appearance of new peaks at 2θ = 38.01°, 44.29°, 64.69°, 77.86°, and 82.14°, which correspond to (1 1 1), (2 0 0), (2 2 0), (3 1 1), and (2 2 2) planes of the face-centered cubic (FCC) lattice of silver [8]. The synthesis of ZnONPs onto this fabric revealed the formation of new peaks at 2θ = 32.21°, 35.09°, 37.05°, 47.61°, 57.33°, and 67.89°. These diffraction patterns correspond to (1 0 0), (0 0 2), (1 0 1), (1 0 2), (1 1 0), and (1 1 2) planes of hexagonal wurtzite ZnO structure, respectively [17,27]. Moreover, Ag diffraction patterns are maintained confirming the high crystalline nature of both particles when incorporated onto flax fabrics.

#### 3.1.4. Attenuated Total Reflectance-Fourier Transform Infrared Spectroscopy (ATR-FTIR)

**Figure 6:**
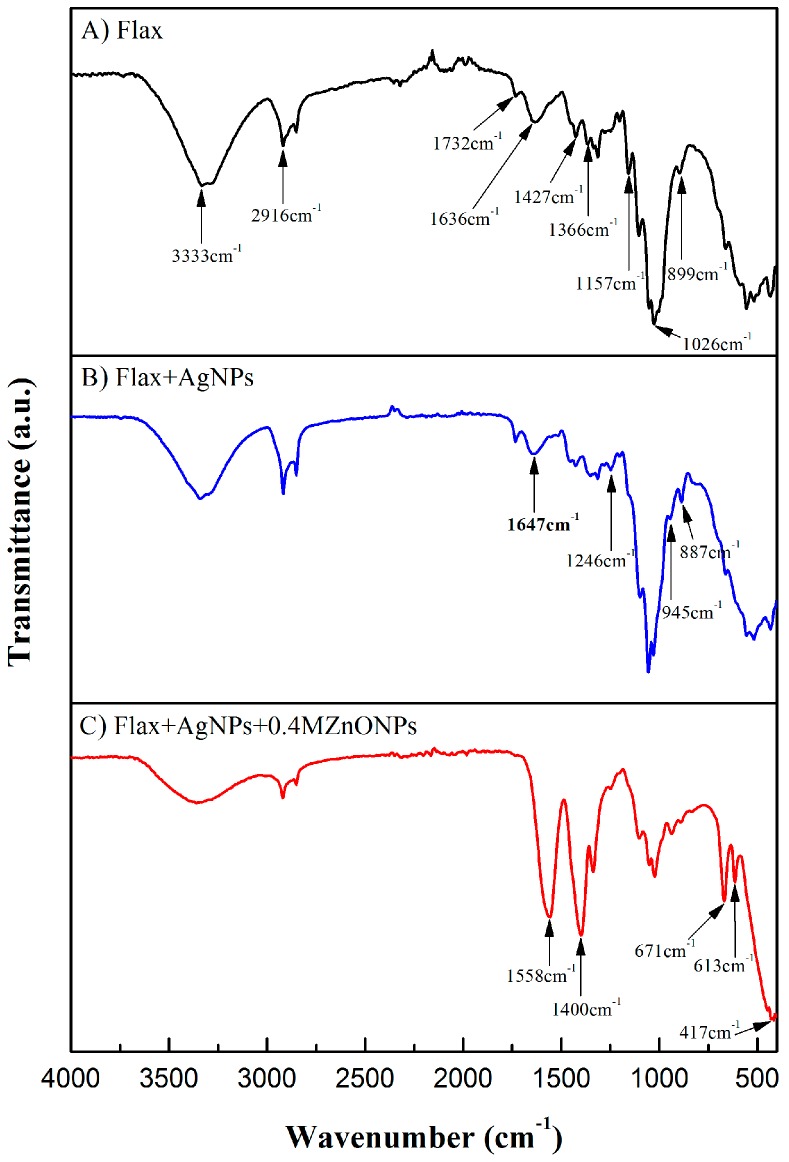
Attenuated Total Reflectance-Fourier Transform Infrared Spectroscopy (ATR-FTIR) spectra of flax fabric (**A**), flax functionalized with AgNPs (**B**), and flax functionalized with AgNPs and ZnONPs (**C**).

The ATR-FTIR spectra of flax, flax coated with AgNPs, and flax functionalized with Ag and ZnO NPs are shown in Figure 6.

The ATR-FTIR spectra of the untreated flax fabric show the typical band peaks of the main constituents of NCF: cellulose, hemicellulose, and lignin. The band at 3333 cm^−1^ corresponds to O-H stretching, which indicates the presence of absorbed water molecules or hydroxyl groups from cellulose and lignin [5,28], the band at 2916 cm^−1^ can be attributed to the asymmetric C-H stretching vibration of cellulose and hemicellulose, the band at 1732 cm^−1^ is related with the C=O stretching vibration of the hemicellulose and lignin, the band at approximately 1636 cm^−1^ represents C=C stretching from lignin, the band at 1427 cm^−1^ was attributed to C-H wagging vibration, the band at 1366 cm^−1^ might be assigned to the C-H bending, the band at 1157 cm^−1^ represented C-O-C vibration, while the band at 1026 cm^−1^ was due to C-O stretch vibration [5,9,29]. 

ATR-FTIR spectra of AgNPs-treated flax fabrics revealed the appearance of several new peaks, which are attributed to PEG, namely 1246, 945, and 887 cm^−1^. Moreover, the band at 1636 cm^−1^ shifted to 1647 cm^−1^, which corresponds to carbonyl group (C=O) formation. Some Ag ions could be reduced to Ag^0^ by the hydroxyl groups from cellulose components of flax fabrics. If this oxidation occurs, then the band correspondent to the carbonyl group (C=O) formation of cellulose will appear at approximately 1647 cm^−1^ [5,30].

Synthesis of ZnONPs onto flax fabrics led to the formation of new peaks. The bands peaking around 1558 and 1400 cm^−1^ are related to asymmetric and symmetric stretching vibrations of C=O group, which may be attributed to ZnONPs precursor (zinc acetate) that was used in the reaction [31]. The appearance of new peaks at 671, 613, and 417 cm^−1^ represents Zn–O stretching vibration, confirming the presence of ZnONPs onto flax fabric surface [6,28,29,32,33]. Since the band peak related with AgNPs formation is very close to the peaks of zinc acetate (precursor), these peaks can mask Ag peaks. Moreover, it is important to refer that FTIR spectrum shown in Figure 6C corresponds to the sample treated with the highest zinc acetate concentration (0.4 M), which originates peaks with higher intensity. In conclusion, all of these findings clearly demonstrate the presence of both Ag and ZnONPs onto the flax fabrics.

#### 3.1.5. Thermogravimetric Analysis (TGA)

TGA analysis was performed in order to estimate the residual weight of the untreated (without NPs) and treated (with NPs) samples, and therefore, to infer about the percentage of NPs deposited on the flax fabric. Flax samples without any treatment were analysed as well as the samples containing 0.1 MAgNPs + 0.2 MZnONPs. After heating up to 600 °C the residues were considered. The amount of NPs deposited on the flax fabric was quantified using equation 2. The flax fabric without NPs revealed 17.72% wt of residues (average value) and the treated samples with NPs exhibited 30.59% wt. While considering equation 2 and assuming that the final sample is 100% composed by flax fabric and NPs, the percentage of NPs onto the sample is approximately 15.6 % wt. (considering the flax fabric residues 17.72% and the sample total residues 30.59%).

### 3.2. Multifunctional Flax Fabrics

The development of fibre-based sensors requires a conductive surface. In fact, the main goal of using AgNPs in this system is to take advantage of their electrical conductivity. The functionalization of NCF with AgNPs reduces the electrical resistivity values of the fibres, as already discussed in a previous research work in which functionalization of jute fabrics with AgNPs decreased the fabrics resistivity values from 1.5 × 10^7^ Ω·m to 1.02 × 10^3^ Ω·m (using 0.1 M precursor concentration) [5]. Therefore, in this work, we decided to use the same concentration of silver precursor in order to obtain conductive flax fabrics and introduce the ZnONPs for the piezoresistive behaviour and other functionalities. In this way, the functionalization of flax fabrics with AgNPs resulted in an electrical resistivity value of 3.33 × 10^3^ Ω·m.

#### 3.2.1. Strain Sensing Mechanism of Flax Fabrics

**Figure 7:**
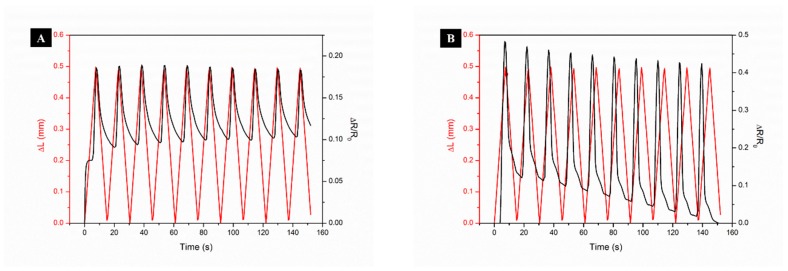

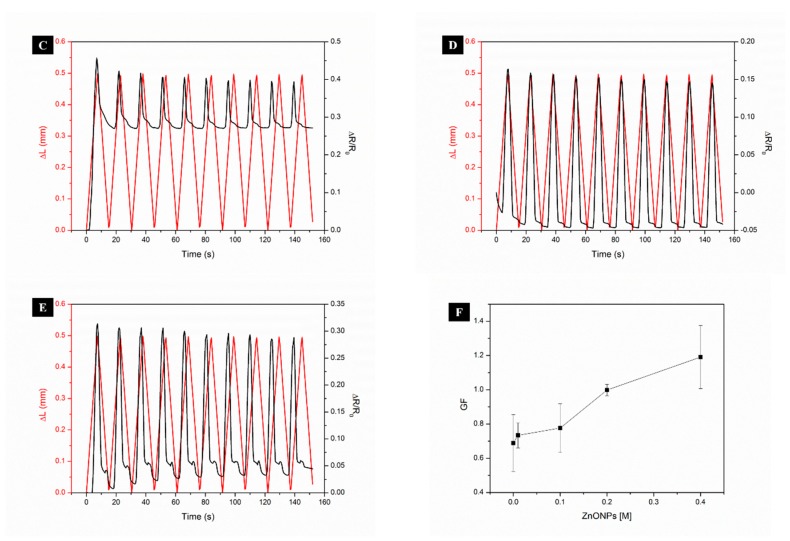
Electromechanical response of flax treated with AgNPs (**A**), flax treated with AgNPs and different ZnONPs precursor concentration from 0.05 to 0.4 M (**B**–**E**), respectively, and gauge factor (GF) values (**F**).

To evaluate the possibilities of using flax fabrics in piezoresistive sensors, Ag/ZnO-modified samples were evaluated by measuring the electrical response under mechanical compression. It has been shown that the percolation threshold corresponds to the region with the largest GF in carbon nanotubes/polymer composites [34], due to the fact that close to the percolation threshold an applied deformation is able to induce strong and reversible variations in the nanotube network configuration (e.g., variations of the nanotubes relative distance) [35], which has a strong influence in the variation of the electrical resistivity. Far from the percolation threshold, network variations are smaller and therefore their effect in the electrical response is also small [36]. Following a similar idea, conducting AgNPs should also reveal strain sensitivity, opening new possibilities to be explored within the field of sensing applications. For that, all of the samples (thickness 1 mm) were compressed 0.5 mm, and the change of the electrical resistance was recorded during 10 loading and unloading cycles. Figure 7 shows the electromechanical response and GF values of flax fabrics, according to the ZnO precursor concentration under use (from 0.05 to 0.4 M).

The application of mechanical pressure onto the Ag/ZnO-treated fabrics causes a reduction in flax thickness, which induces the electro-conductive particles movement, leading to a change in flax electrical resistance [4]. As shown in Figure 7A, flax that was treated with only AgNPs is able to change its electrical resistance under compression. However, the variation on resistance is very small/weak, making this sample not suitable for piezoresistive sensors. On the other hand, doping Ag with ZnO significantly increases the variation in electrical resistance under strain, improving the flax piezoresistive response, leading to an increase of the GF from 0.7 ± 0.2 to 1.2 ± 0.2 (Figure 7F).

The physical phenomenon behind the electromechanical response can be explained by the interactions between the flax fabrics and ZnO NPs fillers. Because of the much higher Young’s modulus of ZnONPs (~127 GPa) [37] as compared to that of the flax fabrics (486 MPa) [38], ZnONPs can be regarded as rigid elements during the strain/release cycles. During the strain cycle, the flax + AgNPs + ZnONPs composite is under compressive stress in the transverse direction of stretching, causing ZnONPs to separate out of the compressive plane and the electrical resistivity to increase. However, for all samples, except the flax + AgNPs + 0.2 MZnONPs, after repeated cycles, a slight drop in both the peak intensity and offset of the maximum of the peak is seen in the next few cycles, due possibly, to stress relaxation. The ∆R/R_0_-time curve seems to be stabilized after a few cycles, which is synchronized with the response of stress under cyclic strain. 

For flax + AgNPs + 0.05 MZnONPs sample, the increase in electrical resistance with applied compression maybe interpreted, as follows. With applied loading, discontinuities in the conductive pathways start to appear within the flax + AgNPs + ZnONPs composite, and the amount of discontinuities increases with an increase of applied load, which results in the increasing of electrical resistance. After releasing the flax + AgNPs + ZnONPs composite to its strain-free condition, most of the disconnected conductive pathways recover to their initial states. However, the some broken AgNPs + ZnONPs result in a permanent contact disruption, which manifested as the increase of overall electrical resistance indicated by the ∆R/R_0_ offset. On the other hand, the sample flax + AgNPs + 0.2 MZnONPs show a reversible electrical resistance and this reversibility is maintained after the 10 strain/release cycles, which means that this amount of ZnONPs could be capable of detecting different movements/pressures.

To evaluate the sensitivity of functionalized flax samples, GF were calculated through the slope of the fraction between the variation in the electrical resistance and the mechanical deformation applied, accordingly with equation number 4 (Figure 7F). From these results, it is clearly visible that the addition of ZnONPs increases the sensor sensitivity, while it also improves the response and recovery time by increasing the charge transport speed. Moreover, the use of the highest ZnO precursor concentration led to higher GF values, increasing the flax fabric sensitivity and making it suitable to be used as piezoresistive textile sensors.

#### 3.2.2. Antibacterial Activity

Several studies report that AgNPs and ZnONPs exhibit remarkable antibacterial activity against a broad spectrum of bacteria [6,8,9,17,39]. Although the mechanisms underlying the antibacterial activity of these NPs are not yet fully understood, the proposed main ones include the disruption of bacterial cell membrane, induction of ROS production leading to oxidative stress, metal ion release (Ag^+^ and Zn^2+^), damage to DNA and proteins, among others [39,40,41,42]. 

**Figure 8:**
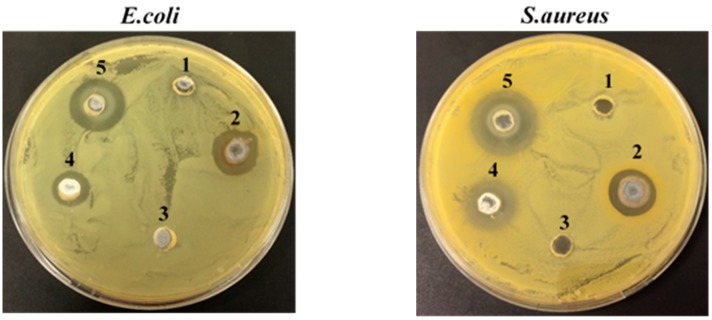
Comparison of inhibition zone of different solutions against *E. coli* and *S. aureus* bacteria. (**1**) PEG; (**2**) AgNPs solution (0.1 M AgNO_3_ with PEG); (**3**) Distilled water; (**4**) ZnONPs solution (0.2 M Zn(CH_3_COO)_2_·2H_2_O with distilled water); and, (**5**) ZnONPs solution (0.4 M Zn(CH_3_COO)_2_·2H_2_O with distilled water).

**Figure 9:**
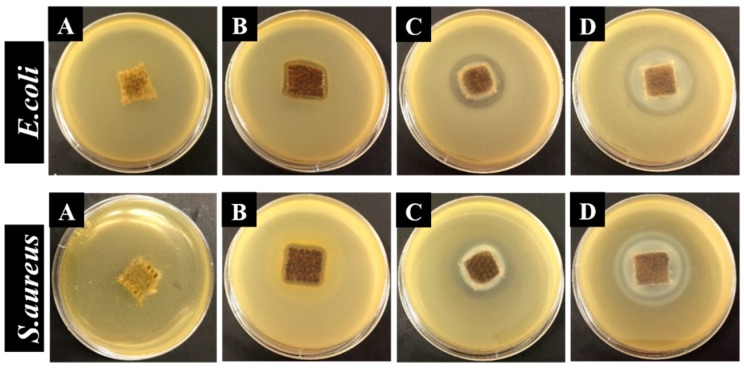
Antibacterial activity of flax (**A**), flax + AgNPs (**B**), flax + AgNPs + 0.2 MZnONPs (**C**) and flax + AgNPs + 0.4 MZnONPs (**D**) for *E. coli* and *S. aureus*.

In the present study, antibacterial activity of NPs solutions (Figure 8) and functionalized flax fabrics (Figure 9) was evaluated against Gram-negative (*E. coli*) and Gram-positive (*S. aureus*). The antibacterial effect was assessed by the formation of a clear inhibition zone (halo) around the samples, which indicates the absence of bacteria growth. Moreover, the size of the inhibition zone, which indicates the antibacterial effect level, was calculated (Table 1) [20]. Firstly, the agar well diffusion method was used to test the antibacterial activity of the solutions containing AgNPs and ZnONPs. The results are shown in Figure 8. 

As shown in Figure 8, the solvents that were used for the synthesis of AgNPs (well 1) and ZnONPs (well 3) show a dense population of bacterial colonies around the samples for both bacteria, indicating no antibacterial effect. On the other hand, the suspensions containing AgNPs (well 2) and ZnONPs (wells 4 and 5) clearly display the formation of an inhibition zone. The inhibition zone size similarity suggests that the antibacterial activity of AgNPs solutions is identical for both *E. coli* and *S. aureus*. In contrast, ZnONPs solutions seem to have a greater antibacterial effect on *S. aureus* as compared to *E. coli*, since the halo diameter is higher for Gram-positive bacteria, which can be explained, even partially, by the difference in composition of each bacteria cell wall [17,43,44]. Another important assumption is that the antibacterial effect of ZnONPs depends on physicochemical properties of NPs, including size, shape, solubility, and the ability to form free biocidal metal ion [39].

As expected, untreated flax fabrics were unable to inhibit the growth of both bacteria tested (Figure 9A). Otherwise, inhibition zones are clearly visible around treated samples (Figure 9B–D), showing the efficacy of both Ag and ZnO NPs as antibacterial agents, when incorporated onto flax fabrics. Interestingly, the addition of ZnONPs onto AgNPs-treated flax induces an improvement in the antibacterial activity of the fabric, as shown by the halo size increase (Table 1). In addition, with the increase of the precursor concentration, the inhibitory effect is stronger against both bacteria. These results show that, besides the antibacterial effect of synthesized NPs, when these NPs are combined onto flax fabrics, the antibacterial properties are maintained, allowing for the production of a natural fibre-based textile with antibacterial properties.

#### 3.2.3. Hydrophobicity Properties

**Figure 10:**
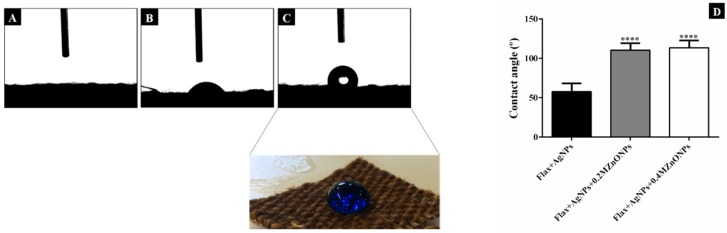
Representative images of water contact angle (WCA) of untreated flax (**A**), flax + AgNPs (**B**) and flax + AgNPs + 0.4 MZnONPs (**C**). Comparison between the WCA values (**D**). Values are represented by mean ± SD. Flax + AgNPs vs. flax + AgNPs + ZnONPs: ****: *p* < 0.0001.

The wettability of the flax fabrics’ surface is dependent of chemical composition and geometrical structure (related with roughness) of the surface [6,24,45]. This property was examined by measuring the WCA in samples’ different locations. Figure 10 shows the images (A-C) and the mean (D) of WCA obtained for untreated flax, flax coated with AgNPs, and flax functionalized with both AgNPs and ZnONPs. If the WCA is smaller or higher than 90°, the surface is considered as hydrophilic or hydrophobic, respectively.

As shown in Figure 10A, the water drops that were released onto untreated flax fabrics were immediately absorbed. Water drops quickly spread on the surface and the samples were completely wetted, resulting in a WCA of 0°. These results were as expected due to the abundance of hydroxyl groups in the cellulose structure of flax fabrics. Moreover, the fabrics pretreatment before the experiments led to a removal of several impurities, including lignin, fat, and waxes contributing to the hydrophilic character of the flax surface [46].

With the addition of AgNPs and ZnONPs, the WCA is continuously increasing, as it can be seen in Figure 10B,C. As previously mentioned, one of the factors that influences the WCA is the surface roughness. Untreated flax fabrics present a smooth surface, as shown in Figure 3B. On the other side, when NPs are incorporated onto fabrics a new roughness is created (Figure 4A). In fact, the synthesis of AgNPs led to higher WCA, demonstrating the increase in the fabrics’ hydrophobicity. Since the PEG polymer is hydrophilic, the increase in WCA is only due to the addition of AgNPs, which contributes to the surface roughness improving the surface hydrophobicity character [8,47]. However, the obtained WCA was around 58°, which means that the fabrics still have hydrophilic properties. This result is consistent with the study of Rajavel et al. [47], which also demonstrates a strong influence of the precursor concentration (AgNO_3_) on the hydrophilic/hydrophobic properties. 

In contrast, the incorporation of ZnONPs induces a significant increase in WCA (higher than 90°), indicating the hydrophobic surface character (Figure 10D). The addition of these NPs produces a uniform coating covering the entire fabric, as shown in Figure 4A (in contrast with the coating with only AgNPs), which contributes to the increase of all surface roughness, leading to higher WCA values. This finding is in agreement with several studies reporting the efficiency of ZnONPs to produce hydrophobic structures [6,11,48]. Therefore, the coating with ZnONPs significantly improves the hydrophobicity of the flax fabric, making it suitable for several applications, such as self-cleaning, easy-cleaning, and water-proof surfaces. 

#### 3.2.4. UV Radiation Resistance and Wash Durability

**Figure 11:**
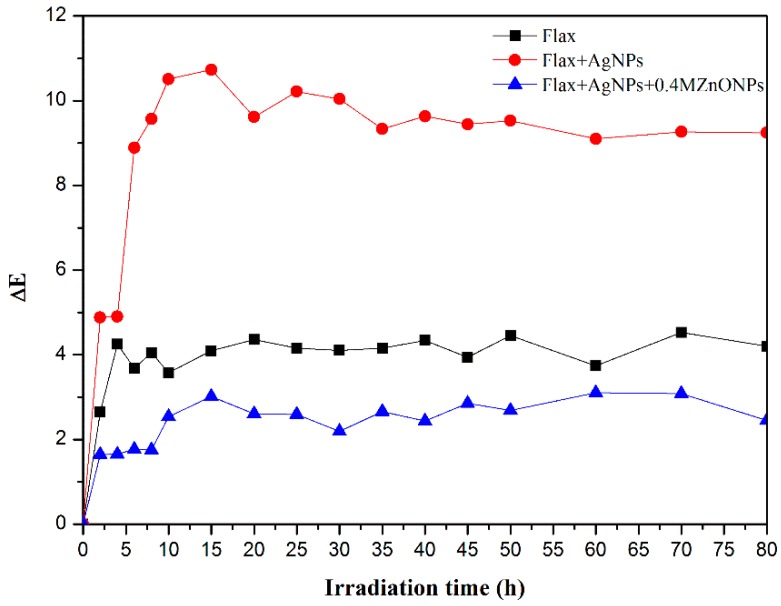
Total colour change with irradiation time of flax fabrics (black curve) flax fabrics coated with AgNPs(red curve) and with Ag/ZnO NPs (blue curve).

UV radiation induces a continuous damage of fibrous materials’ surface, including colour-fading, reduction in mechanical properties, and fibre deterioration [49]. To understand the effect of UV radiation onto fabrics, the monitoring of fabric colour change over time was performed. In this way, the samples were exposed to UV radiation for a total of 80 h, with irradiation intervals ranging from 2, 5 and 10 h, in which the samples’ colour changes were evaluated. Figure 11 shows the overall colour change (∆E) between samples exposed to different times of UV radiation, as compared to the initial samples (time of UV radiation = 0 h), as a function of irradiation time.

From Figure 11 it is clearly visible that flax treated with both Ag/ZnO NPs was the sample with the smallest ∆E values over the total of 80 h of UV radiation, indicating the ability of NPs to avoid UV radiation damage effects onto fabrics.

Regarding the AgNPs-treated flax sample, an abrupt colour difference (∆E) during the first 15 h of irradiation is observed. This is probably due to the continuous formation of AgNPs through the reduction of remaining AgNO_3_. In fact, several studies [5,9] report that UV radiation induces the photoreduction of Ag^+^ ions into Ag^0^ onto the NCF surface. After 15 h of exposure to UV radiation, ∆E values remain relatively identical, suggesting no additional change in colour with an increasing irradiation time.

On the other hand, ZnONPs incorporation onto AgNPs-treated flax promotes a dramatic reduction in ∆E values and this decrease is also observed when compared to untreated flax. These results suggest that ZnONPs can act as UV filtering, resulting in less UV radiation being available to change NCF colour and making this coating more resistant to the aging process [29,49,50,51,52]. Moreover, with the synthesis process of ZnONPs onto AgNPs-treated flax fabrics, the remaining AgNO_3_ was totally removed, preventing the appearance of high initial ∆E values obtained for flax coated with only AgNPs. In conclusion, coating with Ag/ZnO NPs restricted the colour changes observed in the fabrics when exposure to UV radiation.

**Figure 12:**
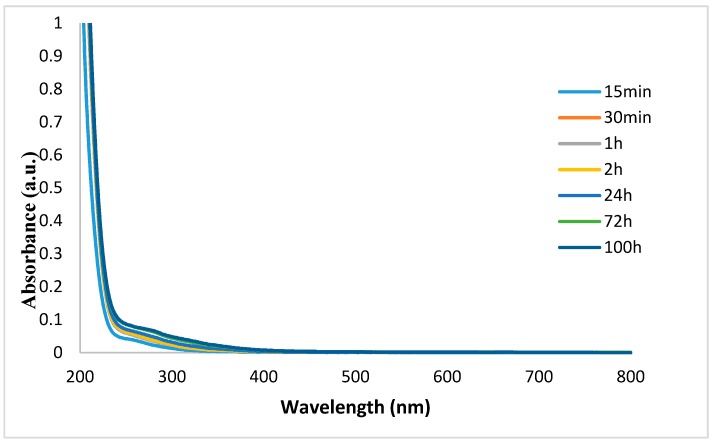
UV-Vis spectra of Ag/ZnO-treated flax washing solutions evaluated at different time points.

To infer about the durability of Ag/ZnO NPs coating, the fibres were immersed in water and subjected to centrifugation. The washing solution was monitored by UV-Vis spectroscopy at different time points: 15 min, 30 min, 1 h, 2 h, 24 h 72 h, and 100 h. Accordingly, with GSDR results from flax functionalized with both NPs (Figure 2), the absorption bands of Ag and ZnO NPs appear at 437 and 380 nm, respectively. As shown in Figure 12, UV-Vis spectra of washing solutions did not present any absorption band at those wavelengths. Moreover, these results were observed for all time points evaluated, demonstrating that both NPs remained attached to the fabric after 100 h of washing. Therefore, these results show the efficient and prolonged NPs adsorption onto fabrics, creating a durable active coating. 

## 4. Conclusions

In this work, for the first time, flax fabrics were functionalized with two different types of nanostructures (Ag and ZnO) to obtain multifunctional smart materials. Ag and ZnO nanostructures were successfully synthetized and adsorbed in flax fabrics, taking into account the sustainability, cost, and simplicity of the methodologies used. GSDR analysis revealed the presence of SPR band of AgNPs peaking at 437 nm and the absorption band at UV region (~380 nm) of ZnONPs. FESEM, EDS, and ATR-FTIR analyses confirmed the synthesis and adsorption of both Ag and ZnO nanostructures onto the flax fabrics surface. The XRD pattern revealed the NPs crystalline structure when incorporated onto flax fabrics. TGA analysis showed that the quantity of NPs deposited onto the flax fabric was 12.87% wt. In order to develop a piezoresistive sensor, a conductive surface was created by functionalizing flax fabrics with AgNPs, which decreased the fabrics resistivity from 1.5 × 10^7^ to 3.33 × 10^3^ Ω·m. Besides AgNPs-treated flax fabric changing their electrical resistance under mechanical compression, the addition of ZnONPs enhanced the piezoresistive behaviour. At the same time, synthesis of ZnONPs led to higher GF values as the ZnO precursor concentration increased, improving the sensor’s sensitivity. Furthermore, the introduction of ZnONPs also improved other properties, including the antibacterial effect against *E. coli* and *S. aureus* and flax fabric hydrophobicity (increasing the WCA above 90°). Simultaneously, functionalized flax fabrics exhibited UV resistance and wash durability. Overall, this work demonstrates the development of multifunctional NCF, which can be used in a variety of monitoring/sensing applications, namely as piezoresistive sensors. Due to the antibacterial activity and hydrophobic character, it is possible to expand the application range of these fibrous systems to several other areas. 

## Figures and Tables

**Table 1:** Inhibition zone diameter of functionalized flax fabrics against *E. coli* and *S. aureus* bacteria accordingly with the JIS L 1902–Halo Method. Values are represented by mean ± standard deviation of triplicates.

Samples	Mean Diameter of the Halos (mm)	
	*E. coli*	*S. aureus*
Flax	0	0
Flax + AgNPs	4 ± 0.08	6.33 ± 0.33
Flax + AgNPs + 0.2 MZnONPs	6 ± 0.22	9 ± 0.28
Flax + AgNPs + 0.4 MZnONPs	14.33 ± 0.09	13.67 ± 0.09
